# Self-management of lower limb lymphedema at home following gynecologic cancer surgery: a qualitative study of women’s experiences and challenges

**DOI:** 10.3389/fpsyg.2025.1728488

**Published:** 2026-01-08

**Authors:** Yuting Tan, Cong Yu, Xiuzhen Mo, Chenghua Sun, Huina Gao, Shufen Song, Qian Zhao

**Affiliations:** 1 Department of Gynaecology, Shenzhen Second People’s Hospital, Shenzhen, Guangdong, China; 2 Department of Nursing, Shenzhen Second People’s Hospital, Shenzhen, Guangdong, China

**Keywords:** gynecological malignancies, home-based nursing care, lower limb lymphedema, qualitative research, self-management

## Abstract

**Introduction:**

As a common chronic complication after gynecological cancer surgery, lower limb lymphedema significantly compromises patients’ quality of life, both physically and psychologically. However, patients’ adherence to home-based CDT, the internationally recommended first-line treatment, was frequently inadequate. This study aimed to explore the lived experiences and core challenges faced by women managing lower limb lymphedema at home after gynecologic cancer surgery. The insights gained can guide the formulation of improved nursing care strategies.

**Methods:**

A descriptive phenomenological design was employed in this study. Semi-structured, in-depth interviews were carried out with participants managing lower limb lymphedema at home after gynecologic cancer surgery. The collected data were analyzed using Colaizzi’s seven-step phenomenological framework. NVivo version 12.0 software was utilized to facilitate the systematic management of the interview transcripts, supporting a rigorous process of coding and thematic extraction.

**Results:**

The final study sample included 10 patients aged from 35 to 75 years. Node integration and refinement led to the construction of a thematic structure comprising 4 parent nodes and 17 child nodes. These 4 central themes about patients’ lived experiences and challenges were elaborated as follows: (1) the burden of illness and psychological adaptation struggles, (2) the knowledge-practice gap in self-care, (3) limitations in social and systemic support networks, and (4) the critical demand for professional nursing support.

**Discussion:**

The process of home-based self-management among patients with lower limb lymphedema after gynecological cancer is complex and influenced by multiple factors, which including physiological, psychological, and social dimensions. Current nursing support systems remain insufficient to meet their actual needs. It is recommended to develop a multi-level intervention model, strengthen family-community-hospital collaborative care mechanisms, and promote digital health tools to enhance patients’ self-management capabilities and quality of life.

## Introduction

Gynecological malignancies represent a major threat to women’s health worldwide. The surgical treatment of these cancers often involves extensive lymph node dissection, particularly in the pelvic and inguinal regions, which predisposes patients to the development of lower limb lymphedema (LLL) (Lindqvist et al., 2017). This complication is characterized by chronic limb swelling, heaviness, and skin fibrosis, significantly impairing patients’ quality of life and potentially leading to secondary complications such as infection, pain, and mobility limitations (Kalemikerakis et al., 2021). Although advances in diagnosis and treatment have significantly improved survival rates, long-term management of postoperative complications remains under-prioritized. Studies have shown that the incidence of LLL among gynecological cancer patients who undergo lymph node dissection ranges from 10 to 30%, with the highest rates observed after cervical cancer surgery (up to 35%), followed by vulvar cancer (approximately 40%), and lower rates following ovarian cancer (10–15%) (Dessources et al., 2020; Kuroda et al., 2017; Lindqvist et al., 2017). In addition, several factors have been identified as contributors to the development or exacerbation of LLL, including obesity, history of radiotherapy, and postoperative infection (Carlson et al., 2020).

Compared with Western countries, research on postoperative LLL among gynecological cancer patients in China is relatively nascent, and epidemiological data remain limited. Preliminary evidence suggests that the incidence of LLL after gynecological cancer surgery in China ranges from 15 to 25%, slightly lower than that reported in Western populations. This difference may be attributed to variations in surgical techniques, extent of lymph node resection, and lack of standardized diagnostic criteria (Daggez et al., 2023). Notably, due to limited public awareness, many patients fail to recognize early symptoms and delay seeking medical attention, resulting in prolonged disease progression and increased individual and societal burden (Koehler et al., 2023). LLL not only causes physical discomfort but also profoundly affects patients’ psychological and social functioning. Body image disturbance, shame, and anxiety are common, and social withdrawal is frequently reported. Some patients even experience depressive symptoms due to the persistent and disfiguring nature of the condition (Kitaw et al., 2024). Moreover, the high cost of compression garments and physical therapy, which are often not covered by health insurance in many regions, imposes a significant financial burden on low-income families (Neuberger et al., 2022).

Currently, there is no curative treatment for LLL, and management primarily relies on Complete Decongestive Therapy (CDT), which includes manual lymphatic drainage, compression therapy, skin care, and exercise (de Sire et al., 2022). In the Chinese context, lymphedema rehabilitation is primarily nurse-led, with limited integration of certified lymphedema therapists or physiotherapists specialized in manual lymphatic drainage (MLD). Although hospital-based CDT programs—which typically deliver manual lymphatic drainage, multilayer bandaging, and education during outpatient visits—provide professional support, most patients must transition to home-based self-management due to the chronic nature of LLL and limited access to specialized services (Zhang et al., 2022). Adherence to long-term self-care practices, such as consistent use of compression garments, proper skin care, and regular physical activity, is essential for disease control (Zeng et al., 2024). However, studies have shown that patients face multiple challenges in the home setting, including fragmented information, insufficient skill acquisition, financial constraints, and lack of family support, all of which contribute to poor adherence, recurrent swelling, and suboptimal outcomes (Moffatt et al., 2019; Yin et al., 2025; Ali et al., 2025). These issues present significant barriers to the process and efficacy of self-managed rehabilitation for patients with lymphedema in the home setting (Bakar and Tuğral, 2017; Abboud et al., 2021). Therefore, there is an urgent need to establish a systematic and individualized home-based nursing support system to enhance patients’ self-management capacity and improve their quality of life. This study aims to explore the lived experiences of Chinese women with LLL following gynecological cancer surgery, and to identify core challenges and needs in the context of home-based self-care. The findings will provide a theoretical foundation for developing culturally appropriate nursing interventions.

## Materials and methods

Phenomenological analysis was employed in this qualitative study. Descriptive phenomenology, is particularly suited to exploring the essence of lived experiences among individuals facing a poorly understood chronic condition. Given our aim to capture the first-person, everyday realities of women managing lower limb lymphedema at home—rather than to generate theory or test hypotheses—this approach aligns with the epistemological stance of bracketing preconceptions and foregrounding participants’ voices. This contrasts with interpretive phenomenological analysis (IPA), which emphasizes researcher interpretation, and grounded theory, which seeks explanatory models. Individual in-depth interviews were conducted using a semi-structured interview outline, and Colaizzi’s 7-step analysis was used to organize and analyze data. The interview outline was designed to uncover the deep meanings and essential structures of their self-management experiences at home with lower limb lymphedema after gynecological cancer surgery.

### Study participants

A maximum variation purposive sampling strategy was employed to ensure diversity in age, disease duration, educational background, and cultural context. Participants were recruited from the Lymphedema Rehabilitation Clinic and Gynecological Ward of Shenzhen Second People’s Hospital between August 2024 and August 2025. Sample size was determined by the principle of data saturation, defined as the point at which no new themes emerged across three consecutive interviews. The inclusion criteria were as follows: ① Histopathologically confirmed diagnosis of gynecological malignancy (cervical, endometrial, ovarian, or vulvar cancer) after curative surgery; ② LLL diagnosis required ≥2 cm interlimb circumference difference at one or more standardized measurement points (ankle, calf, thigh) or clinical confirmation by a certified lymphedema therapist using the International Society of Lymphology (ISL) staging system (Stage I: pitting edema; Stage II: non-pitting, tissue fibrosis; Stage III: elephantiasis with skin changes). Imaging (e.g., lymphoscintigraphy) was used only when diagnosis was uncertain, ③ Participants must have been managing their lymphedema at home for a minimum duration of 1 months following completion of the intensive decongestion phase; ④ Adequate cognitive and communication abilities to participate in the in-depth interviews; ⑤ Willingness to provide written informed consent and participate voluntarily. The exclusion criteria included: ① Severe comorbidities that could confound limb swelling assessment (e.g., active infection, deep vein thrombosis, heart failure, or renal insufficiency); ② Secondary lower limb edema caused by non-surgical factors such as cardiac, hepatic, renal, or venous insufficiency.

### Develop an interview outline

Semi-structured in-depth interviews were conducted to explore participants’ self-management experiences and support needs in the home setting. An interview guide was developed based on the study objectives and a comprehensive literature review, structured around five thematic domains to ensure both openness and systematic inquiry. The guiding questions are as follows: ① How did you first learn about lymphedema? How would you describe your current level of knowledge and skill in managing it? What aspects did you feel were lacking? ② How did you currently managed your condition at home? What challenges have you encountered? Have you ever interrupted your self-care routine, and if so, why? ③ How did you perceived your current physical condition compared to before your illness? What changes have you noticed? ④ Who or which institutions have provided you with support? In your view, what are the current gaps in follow-up care and rehabilitation guidance within the healthcare system? ⑤ What kind of support did you most hope to receive from healthcare providers? From a patient’s perspective, what suggestions would you offer for improving care?

### Data collection method

A pilot interview (*n =* 2) was conducted to refine question wording, sequence, and probing techniques, ensuring clarity and flow. All formal interviews were carried out by two trained interviewers, both master’s-level nurses with clinical experience and formal training in qualitative research. They received standardized training in empathetic listening, clarification, and non-judgmental responses to minimize bias. The two interviewers were not involved in the clinical care of any participant; they were research nurses with no prior therapeutic relationship with the patients, thereby reducing social desirability bias. To mitigate researcher bias, both interviewers maintained reflexive journals documenting their assumptions and emotional responses during data collection, and these were reviewed during team debriefings prior to analysis. Interviews were conducted in private consultation room of the lymphedema rehabilitation clinic, Shenzhen second people’s Hospital, each lasting between 40 and 90 min, with flexibility based on participants’ comfort and fatigue levels. Each participant underwent one to two interviews to ensure depth and richness of data. Participants were encouraged to express themselves in their own words, with researchers intervening only to clarify or probe when necessary. All interviews were audio-recorded with participant consent and transcribed verbatim within 24 h. Transcripts were anonymized using participant codes (e.g., P1, P2). All data were used solely for research purposes and will be securely destroyed upon study completion in accordance with institutional guidelines.

Main sources of social support were defined as primary individuals providing regular assistance related to lymphedema management. According to the MOS social support survey framework, social support categories included: (1) Spouse/partner; (2) Children; (3) Parents; (4) Extended family; (5) Healthcare professionals; (6) Patient peer groups; (7) Community organizations; and (8) None. Two researchers independently extracted support provider mentions from transcripts, with discrepancies resolved through consensus. The primary source for each participant reflected their most consistently referenced support provider across multiple contexts of daily lymphedema management.

To uphold data integrity, multiple strategies were employed. Prior to data collection, all necessary materials were confirmed to be operational. Data triangulation was achieved through concurrent audio recording, note-taking, and observation during interviews. After each interview, recording clarity and authenticity were verified, and materials were cataloged and secured without delay. The study was approved by the Ethics Committee of Shenzhen Second People’s Hospital (Approval no. 2025-700-01YJ).

### Data analysis

To enhance the reliability and validity of the data analysis, this study implemented cross-checking and independent analysis procedures. Interview transcripts were verbatimly prepared within 24 h post-interview and subsequently reviewed by a second researcher to verify their accuracy and completeness. Two researchers independently analyzed the data using Colaizzi’s seven-step phenomenological method. Coding and categorization were facilitated using NVivo 12.0 software (QSR International, Doncaster, Australia). Any discrepancies in the analysis were resolved through consultation and discussion within the research team.

The data were analyzed following Colaizzi’s seven-step method: ① Familiarization: Repeated reading of all interview transcripts; ② Identifying Significant Statements: Extraction of relevant statements. ③ Formulating Meanings: Deriving meaning from the statements; ④ Clustering Themes: Grouping meanings into thematic clusters; ⑤ Developing Exhaustive Description: Writing a detailed description of the phenomenon; ⑥ Producing Fundamental Structure: Distilling the core structure of the experience; ⑦ Seeking Verification: Returning the findings to participants for confirmation. As part of the verification step (Colaizzi’s Step 7), summary sheets of emergent themes were returned to the 10 participants via telephone or WeChat. All provided confirmatory feedback, two suggested minor wording refinements (e.g., replacing ‘helplessness’ with ‘frustration’), and one expressed that the theme ‘financial strain’ underrepresented the emotional toll of cost-related decisions. These inputs were integrated into the final thematic formulation.

In practice, transcripts were produced within 24 h post-interview. The coding process involved reviewing nodes and grouping them into themes based on semantic similarities and conceptual relationships. The strength of the thematic identification was ensured by continuously cross-referencing the categories with the original data.

## Results

### Participants’ baseline characteristics

A total of 10 patients with lower-limb lymphedema after gynecological cancer surgery were included in this study ranged age from 35 to 75 years, with a mean age of 50 years. Detailed baseline characteristics of the participants are presented in Table 1. All participants had developed varying degrees of lower-limb lymphedema after surgery, with symptom duration ranging from 6 months to 5 years.

**Table 1:** Basic characteristics of participants.

Participant ID	Age (years)	Education level	Occupation	Cancer type	Time since surgery (months)	Chemotherapy (Yes/No)	Radiotherapy (Yes/No)	Duration of LLL (months)	Lymphedema stage	Current self-management practices	Main sources of social support	Medical insurance
P01	68	Junior High School	Unemployed	Cervical Cancer	78	Yes	Yes	28	Stage III	Medication^1^ management, dietary control, manual lymphatic drainage, wearing compression stockings	Children	Rural Medical Insurance
P02	58	Junior High School	Retired	Cervical Cancer	39	Yes	Yes	31	Stage II	Functional exercises, wearing compression stockings, bandage compression	Spouse or Partner	Urban Medical Insurance
P03	53	High School	Accountant	Endometrial Cancer	70	Yes	Yes	14	Stage III	Manual lymphatic drainage, wearing compression stockings, functional exercises	Spouse or Partner, Patient Groups	Urban Medical Insurance
P04	59	Junior High School	Retired	Ovarian Cancer	51	Yes	No	41	Stage II	Dietary control, traditional Chinese medicine regulation	Spouse or Partner, Community Organizations	Urban Medical Insurance
P05	54	Junior High School	Self-employed	Ovarian Cancer	46	Yes	No	12	Stage III	Wearing compression stockings, bandage compression	Spouse or Partner, Medical Staff	None
P06	60	High School	Retired	Endometrial Cancer	67	Yes	Yes	6	Stage II	Wearing compression stockings, manual lymphatic drainage	Children	Rural Medical Insurance
P07	45	Bachelor’s Degree	Sales	Ovarian Cancer	108	Yes	Yes	38	Stage III	Wearing compression stockings, bandage compression, psychological adjustment	Parents	Urban Medical Insurance
P08	53	Junior High School	Unemployed	Cervical Cancer	30	Yes	Yes	26	Stage III	Wearing compression stockings, bandage compression	No Support	None
P09	62	Junior High School	Retired	Endometrial Cancer	38	Yes	No	15	Stage II	Manual lymphatic drainage, wearing compression stockings, functional exercises	Children, Community Organizations, Patient Groups, Medical Staff	Urban Medical Insurance
P10	75	Bachelor’s Degree	Retired	Cervical Cancer	96	Yes	No	32	Stage III	Wearing compression stockings,	Children	Urban Medical Insurance

^1^Refers to occasional symptomatic use of medications for comorbidities or secondary symptoms (e.g., NSAIDs for pain, topical agents for skin changes).

**Figure 1:**
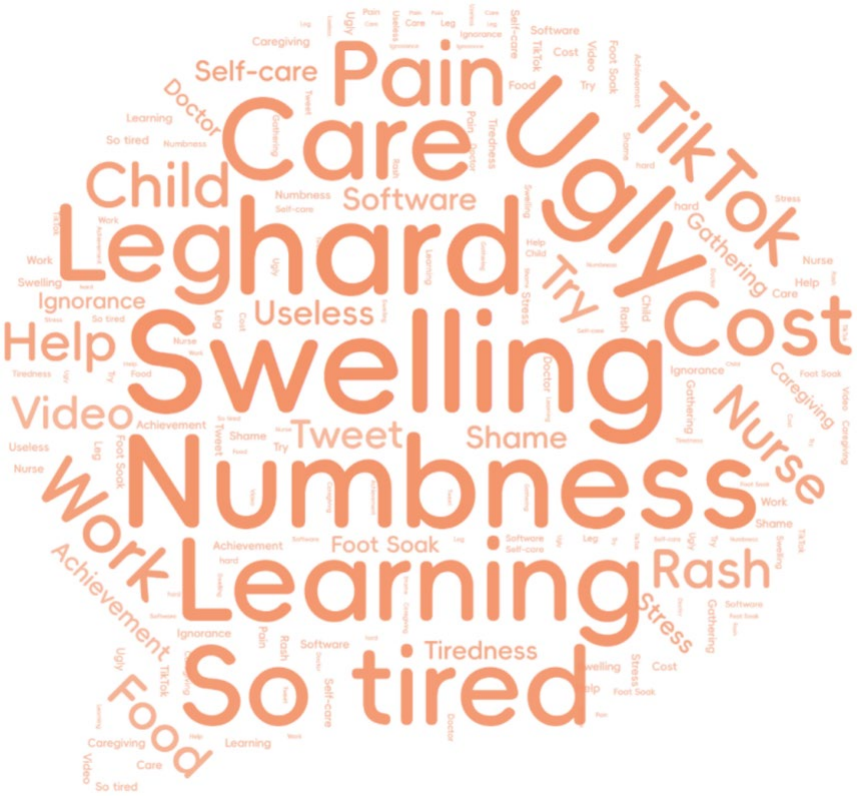
Word cloud visualization of high-frequency terms (>15 occurrences) from interview transcripts.

To visualize prominent themes in the transcript data, a word cloud was generated using the word frequency function in NVivo 12.0, wherein the size of each word corresponds to its frequency (Figure 1). The prominence of terms such as “Swelling,” “Numbness,” and “Pain” directly reflects the core physical symptoms that patients consistently described as central to their daily experience. The large size of “Ugly” and “Shame” visually illustrates the profound psychological impact of lymphedema on body image, while “Cost” and “So tired” highlight the significant economic and emotional burdens. The appearance of terms like “Care,” “Self-care,” “Nurse,” and “Doctor” underscores the importance of healthcare relationships in patients’ management experiences. The presence of “Learning,” “Video,” and “TikTok” suggests patients’ interest in and engagement with various educational resources, which aligns with our finding about the knowledge-practice gap in self-care. This word cloud served as an analytical starting point that guided our subsequent coding process, helping us identify key areas requiring deeper exploration during the formal thematic analysis.

**Figure 2:**
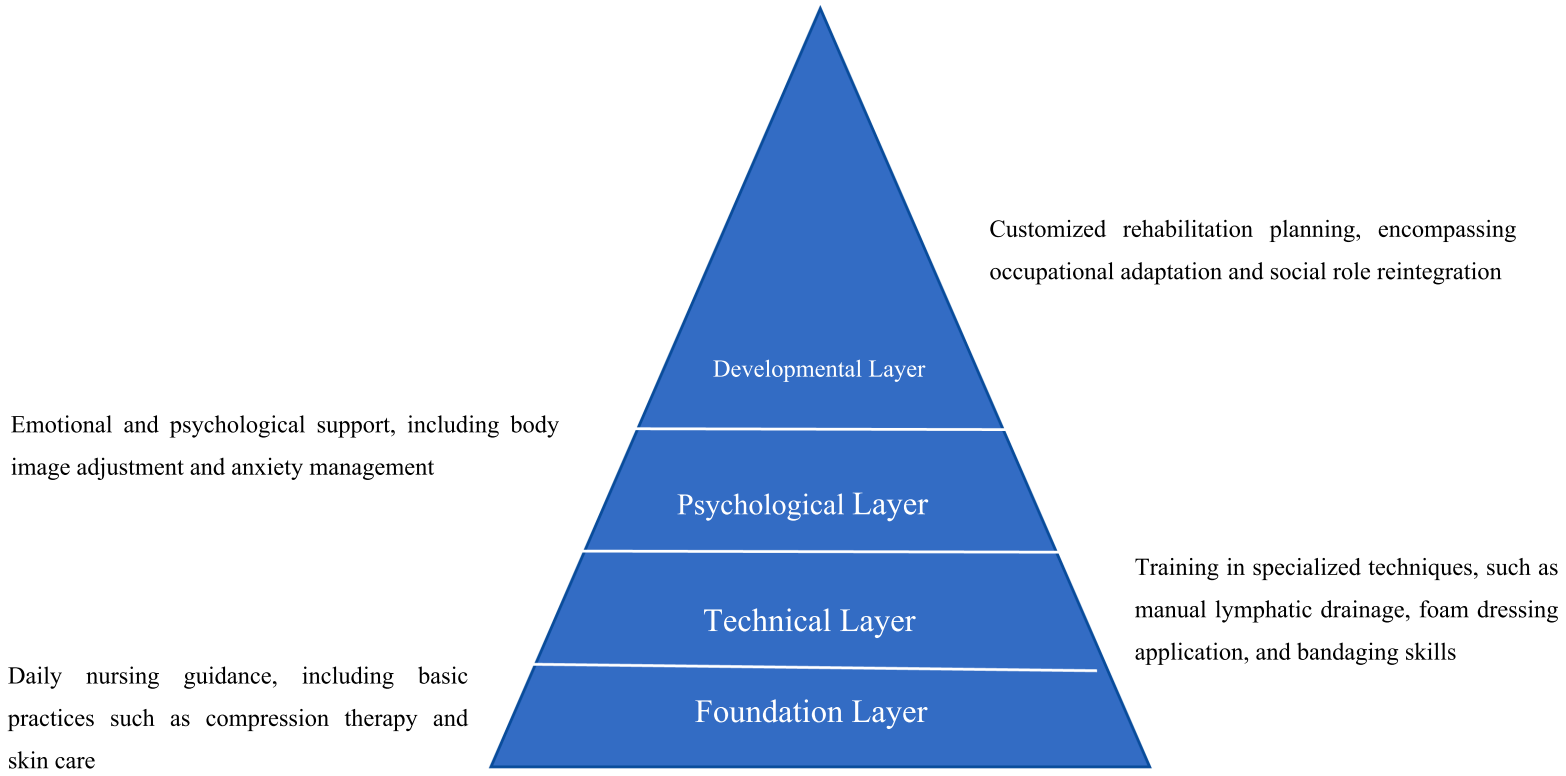
Hierarchical model for specialized nursing needs of patients with lower extremity lymphedema.

Subsequent node integration and refinement led to the construction of a thematic structure comprising 4 parent nodes (themes) and 17 child nodes (sub-themes). The complete hierarchy of nodes and their corresponding reference points is detailed in Table 2. These 4 central themes about patients’ lived experiences and challenges were elaborated as follows: (1) the burden of illness and psychological adaptation struggles, (2) the knowledge-practice gap in self-care, (3) limitations in social and systemic support networks, and (4) the critical demand for professional nursing support (Figure 2).

**Table 2:** Themes, number of interview materials, reference points and supporting quotes.

Themes and sub-themes	Number of node material sources^a^	Reference points^b^	Supporting quotes
1. Theme: the burden of illness and psychological adaptation struggles	9	41	
1.1 Anxiety and Uncertainty About Disease Progression	6	12	“Every morning I check my leg. If it looks even a bit larger, I spend the whole day worrying that it’s getting worse and there’s nothing I can do.”
1.2 Financial Strain from Out-of-Pocket Treatment Costs	4	11	“The compression stockings wear out every few months, and they are so expensive. It’s a constant financial pressure on top of all the other medical bills.”
1.3 Loss of Autonomy and Dependence in Daily Activities	3	8	“I used to be the one who took care of my family. Now I have to ask my husband to help me put on my shoes. It makes me feel like a burden.”
1.4 Distress from Altered Body Image and Social Withdrawal	4	10	“I’ve stopped wearing skirts and avoid social gatherings. I cannot bear the thought of people asking me what’s wrong with my leg.”
2. Theme: the knowledge-practice gap in self-care	7	56	
2.1 Deficits in Practical Skills for Compression Therapy	6	15	“The therapist bandaged my leg perfectly in the clinic, but when I try to do it myself at home, it’s either too loose and does nothing or so tight it cuts off my circulation.”
2.2 Challenges in Selecting and Using Compression Garments	7	15	“I spent so much money on different stockings, guessing my size. One pair was impossible to get on, and another rolled down my thigh all day. It’s frustrating and wasteful.”
2.3 Reliance on Unverified and Misleading Online Information	4	9	“One website said to elevate my leg, another said to exercise, and a blog recommended a special herbal patch. I tried them all, but nothing worked, and I just felt more confused.”
2.4 Lack of Actionable and Individualized Health Education	3	8	“They told me ‘maintain a healthy weight’ and ‘do these exercises,’ but they never showed me how to adapt the exercises for days when my leg is particularly heavy and painful.”
2.5 Physical Discomfort as a Barrier to Consistent Adherence	3	9	“The pressure garment is so hot and itchy, especially in summer. Sometimes I just have to take it off for a few hours to feel normal again, even though I know I should not.”
3. Theme: limitations in social and systemic support networks	5	37	
3.1 Insufficient Practical Care Skills from Family Members	5	13	“My wife wants to help with the massage, but she’s terrified of applying too much pressure. We both end up feeling stressed and helpless.”
3.2 Lack of Time and Emotional Support from Immediate Family	5	10	“My son says, ‘Just do what the doctor said,’ but he does not understand the daily struggle. I do not want to complain all the time and become a source of stress for them.”
3.3 Absence of Sustained Community-Based Peer Support	3	5	“We had a chat group, but it slowly died out. Everyone was struggling alone again. It would be nice to have a regular meeting to share our ups and downs.”
3.4 Geographic and Resource Barriers to Accessing Professional Care	4	9	“The specialist clinic is a three-hour bus ride away. For a 30-min appointment, I lose almost my entire day. It’s just not sustainable for regular check-ups.”
4. Theme: the critical demand for professional nursing support	9	54	
4.1 Urgent Need for Standardized, Home-Based Nursing Guidance	7	23	“We need a clear, step-by-step plan for managing flares at home. What do I do if my skin gets red? Who do I call? Right now, it feels like we are just left to figure it out on our own.”
4.2 Demand for Visual and Accessible Educational Materials	8	13	“A demonstration video I can pause and rewatch would be so much better than a leaflet. Seeing how to properly wrap the bandage would build my confidence.”
4.3 Desire for Proactive and Continuous Professional Follow-up	9	10	“It would be a huge relief if a nurse called me once a month to check in. Just knowing that someone is tracking my progress would make me feel supported and less alone in this.”
4.4 Call for Integrated Hospital-Community Care Transition	7	9	“When I left the hospital, it felt like a cliff edge. There was no handover to my local clinic. I fell through the cracks, and my condition worsened before I managed to get another specialist appointment.”

Remarks: “a” refers to the number of interview materials containing the node; “b” refers to the source of the node in all interview materials. The green shades are the “themes,” and the white shades represents the “sub_themes” part.

### Burden of illness and psychological adaptation struggles

Patients with lower-limb lymphedema following gynecological cancer surgery face a lifelong physical and emotional burden, where ongoing symptom management intersects with profound psychological distress. On one hand, they must contend with persistent physical symptoms and the demanding routine of daily self-care; on the other, they experience heightened emotional strain and a sense of social isolation, complicating their ability to adapt. As one participant expressed:

“The first thing I do every morning is check if my leg has improved. It’s so depressing—I do not even want to go outside.” (P4).

This internal conflict between physical reality and emotional well-being evolved across different stages of illness. In the early phase, denial and avoidance were common coping mechanisms. Over time, as patients gradually accepted their condition, they began to develop adaptive strategies. One participant reflected:

“Now I’m more emotionally stable. Even if my leg swells after walking too much, I know to rest immediately and apply higher compression when bandaging.” (P6).

These shifts illustrate a dynamic process of psychological adjustment shaped by prolonged exposure to chronic symptoms and caregiving demands.

### Gap between knowledge acquisition and practical competence

A significant discrepancy exists between patients’ access to self-management information and their ability to apply it effectively. While participants actively sought knowledge through online searches and peer exchanges, the information obtained was often fragmented, inconsistent, and lacking scientific validation. At the same time, the absence of structured professional guidance undermined their confidence and self-efficacy.

One patient shared:

“I searched online for ways to reduce leg swelling—like wearing compression garments—so I used my old varicose vein stockings. After wearing them for a while, I did not notice any real improvement.” (P3).

Another reported a more severe consequence:

“I went to a traditional Chinese massage clinic. The therapist recommended massage, acupuncture, and cupping. My swelling improved at first, but half a month later, I suddenly developed a fever over 40 °C, and my leg became hot and swollen again.” (P4).

This disconnect between information and practice frequently led to inappropriate or harmful self-care behaviors. For instance:

“My leg is not just swollen—it’s numb and painful. I heard camphor oil improves circulation, so I applied it along with some blood-activating ointments and patches. Instead, my skin got worse and more irritated.” (P7).

Moreover, selecting appropriate medical devices posed additional challenges:

“Choosing compression stockings was all trial and error. I did not know which brand was suitable. Even with the same Class II gradient pressure, different brands felt completely different. Finding the right one was incredibly difficult—and expensive.” (P8) These accounts highlight how inadequate knowledge translation contributes to mismanagement and increased patient vulnerability.

### Limitations of social support networks

Participants’ social support systems were marked by fragmentation and insufficient professional involvement. Family members, though often willing, lacked the necessary expertise to provide effective care. Meanwhile, limited access to community-based or specialized services further constrained recovery and long-term management.

As one patient noted:

“My children are working, and my husband is afraid to touch my leg. Most of the time, I have to manage everything on my own.” (P10).

Geographic disparities exacerbated these challenges. In rural and county-level areas, healthcare facilities typically lacked dedicated lymphedema services, while tertiary hospitals were often overburdened. A participant explained:

“There’s no lymphedema service at the nearby community health center. Going to the hospital is exhausting for us.” (P1).

Another described the strain on specialist resources:

“One lymphedema nurse sees over 10 patients in a single morning session. Manual lymphatic drainage lasts only about 15 min, and scheduling regular treatments is complicated.” (P6).

These systemic gaps left patients feeling isolated and underserved, particularly those without access to consistent, expert-led care.

### Professional nursing care as a key to enhanced self-management and quality of life

Personalized, evidence-based nursing interventions were consistently identified as pivotal in improving both self-management capabilities and overall quality of life. Participants expressed a strong desire for comprehensive, standardized education covering compression therapy, exercise regimens, skin care, and early complication recognition.

One participant emphasized:

“I really hope hospitals could offer systematic nursing guidance tailored to our needs.” (P7).

Furthermore, there was notable interest in innovative, accessible models of care delivery. As another participant envisioned:

“If AI-powered tools could monitor my limb circumference and guide treatment remotely, elderly patients like me could receive professional support without leaving home—that would be ideal.” (P10)

## Discussion

This study employed a phenomenological approach to explore the self-management experiences and core needs of patients with lower limb lymphedema following surgery for gynecological malignancies in home-based settings. Through Colaizzi’s seven-step analysis method, in-depth interviews with 10 patients were systematically coded, leading to the construction of a thematic structure comprising 4 parent nodes and 17 child nodes. The findings reveal the complex challenges faced by patients during long-term self-management, which extend beyond physical symptoms and difficulties in self-care practices to include psychological adjustment, reconfiguration of social relationships, and barriers in accessing resources. The study further indicates that insufficient disease knowledge and a lack of nursing skills commonly hinder patients’ self-management efficacy. Many participants relied on fragmented information sources—such as online searches and peer communication—yet often struggled to distinguish evidence-based guidance, resulting in inappropriate self-care behaviors. Moreover, limited support capabilities among family members and uneven distribution of community and healthcare resources increased the risk of discontinuities in care.

### Multidimensional analysis of the conflict between disease burden and psychological adaptation

This study reveals significant psychological adaptation challenges faced by patients with lower limb lymphedema following surgery for gynecological malignancies. These challenges arise not only from alterations in physical appearance but also involve deeper identity conflicts and the renegotiation of social roles. Participants commonly exhibited anxiety related to body image distortion and cognitive dissonance due to the chronic and irreversible nature of their condition—a finding consistent with Abakay et al.’s concept of “body alienation” among lymphedema patients (Abakay et al., 2023).

Furthermore, family support played a critical yet complex role in psychological adaptation. While 60% of respondents reported receiving emotional support from family members in daily life, only 10% believed their caregivers possessed adequate skills to assist in lymphedema rehabilitation. This observation resonates with Jabir et al.’s findings, which suggest that although emotional support from family can significantly reduce anxiety, unskilled caregiving may inadvertently increase patients’ psychological burden (Jabir et al., 2025). Therefore, implementing a family-system intervention model in clinical nursing practice is recommended. Such an approach would involve family members in structured psychological support to enhance patients’ overall adaptive capacity. Programs like the Family Empowerment Program, introduced by Rashidi et al. (2025), while providing patient education, it is crucial to enhance nursing skills training for caregivers to build a more effective support network.

### Mechanisms underlying the gap between knowledge acquisition and practical skills

A significant disparity exists between patients’ acquisition of self-management knowledge and their ability to apply it in practice. On one hand, patients often obtain information through channels such as internet searches and peer communication, yet this information tends to be fragmented and lacking scientific rigor (Jabir et al., 2025). On the other hand, the absence of systematic professional guidance results in reduced self-efficacy. Specifically, deficits in expert instruction regarding diet, exercise, and compression garment use contribute to diminished confidence in self-management—a finding consistent with Wang et al. (2025) who reported a strong positive correlation (r = 0.72, *p* < 0.01) between knowledge mastery and self-management ability among lymphedema patients.

This study further identified online search as a primary information source (Participants 3, 5, 9, 10), albeit with considerable risks of misinformation. Koly et al. (2025) noted that most of lymphedema patients (*n =* 28) relied heavily on internet searches, yet 62% were unable to distinguish commercial promotions from evidence-based recommendations (Koly et al., 2025). Moreover, AI-driven algorithms can exacerbate information bias (Shen et al., 2025). Common misconceptions observed included “soaking feet improves circulation” (P3) and “applying traditional Chinese herbal patches reduces swelling” (P7)—misguided practices that may potentially worsen the condition.

Our findings also reflect culturally embedded care expectations in China. Participants frequently expressed reluctance to ‘burden’ family members (e.g., P10: ‘I do not want to complain… become a source of stress’), aligning with Confucian values of familial harmony and stoic endurance. Additionally, the preference for traditional remedies (e.g., herbal patches, cupping) observed in P4 and P7 reflects a broader societal trust in complementary medicine, sometimes at the expense of evidence-based care. These culturally shaped behaviors warrant culturally tailored interventions that validate patients’ health beliefs while guiding safe self-care practices.

In this study, multiple participants expressed a strong desire for the development of an “intelligent wearable monitoring system” that could provide real-time feedback on garment fit and pressure levels via embedded sensors. Such a system has the potential to enhance self-management capabilities, treatment adherence, and psychological well-being, thereby improving the quality of home-based care. The authors recommend developing a mobile health (mHealth) application incorporating features such as dynamic risk assessment, personalized reminders, video-based corrective feedback, and a tiered discussion forum—aligning with the mHealth intervention framework proposed by Choolayil et al. (2025).

### Construction of a hierarchical model for specialized nursing needs

Based on a systematic analysis of patient needs, this study proposes a “Pyramid Model” for lymphedema care needs, grounded in Maslow’s Hierarchy of Needs. This model consists of four distinct levels, offering a theoretical framework for the design of nursing services for lower limb lymphedema: (1) Foundation Layer: Daily nursing guidance, including basic practices such as compression therapy and skin care; (2) Technical Layer: Training in specialized techniques, such as manual lymphatic drainage, foam dressing application, and bandaging skills; (3) Psychological Layer: Emotional and psychological support, including body image adjustment and anxiety management; (4) Developmental Layer: Customized rehabilitation planning, encompassing occupational adaptation and social role reintegration.

Our proposed pyramid model finds strong empirical validation in emerging stepped care frameworks. Peters primarily evaluated the safety and effectiveness of the “Walk ‘n Watch” structured progressive exercise program in the rehabilitation of hospitalized stroke patients in Canada. This was a phase 3, multi-center, pragmatic, cluster-randomized stepped-wedge designed randomized controlled trial that validated the effectiveness of progressive rehabilitation protocols (Peters et al., 2025). Rossi established through their randomized controlled trial that stepped care frameworks effectively balance intervention intensity with patient needs—a principle directly applicable to lymphedema management. The authors found that dynamically adjusting support intensity based on symptom severity and self-efficacy scores improved adherence by 28% compared to static care models (Rossi et al., 2025). Similarly, Yang conducted a comprehensive scoping review analyzing implementation outcomes across 27 cancer-related stepped care programs, identifying that tiered approaches with clear transition protocols between care levels optimized resource utilization while maintaining quality of care. Their analysis demonstrated a 35% reduction in unnecessary specialist referrals when primary care providers were equipped with clear decision pathways (Yang et al., 2024). These findings substantiate our recommendation for a phased approach to lymphedema care that escalates or de-escalates professional involvement based on objective indicators of disease control and patient capability.

Furthermore, there is a critical need to establish standardized knowledge dissemination mechanisms to prevent misconceptions resulting from fragmented information. Health education materials for lower limb lymphedema should balance scientific accuracy with accessibility, using patient-friendly formats and language. It is also advisable to expand publicity channels, particularly through new media platforms, to broaden the reach of health education and enhance public awareness of lymphedema (Li et al., 2025).

Studies indicate that the suboptimal effectiveness of self-care among lymphedema patients at home can be attributed to multiple factors, including insufficient professional support (Mwesigye et al., 2025), lack of tailored educational materials (Guan et al., 2025), limited adoption of remote technologies (Mullan et al., 2025), fragmented interdisciplinary collaboration (Hsu et al., 2024), and economic barriers (Yarmohammadi et al., 2024). In response, we propose the establishment of a three-tiered support network integrating family, community, and hospital resources. Such a collaborative framework could systematically address issues such as educational gaps, resource fragmentation, and discontinuities in follow-up care. Future research should further evaluate the cost-effectiveness of this model across different healthcare systems. For example, the Dutch “Integrated Lymphedema Care Model,” which incorporates smart bandaging technology and close coordination among family, community, and hospital providers, has been shown to improve patients’ self-management capabilities by 40% (Tümkaya and Seven, 2025).

### Study limitations

This study has several limitations. The primary sample was recruited from healthcare institutions in Shenzhen, which may introduce regional bias. Future studies should include participants from broader geographical areas to improve the generalizability of the findings. Additionally, the under representation of patients with severe lymphedema (Stage III and above) highlights the need for further investigation into the specific needs of end-stage populations.

Although interviewers were trained to remain non-judgmental, their professional identity as nurses may have subtly influenced participants’ willingness to disclose negative experiences with the healthcare system. Future studies could involve interdisciplinary interviewers (e.g., psychologists or sociologists) to enhance perspective diversity.

## Conclusion

The significance of this study lies in its patient-centered approach, which uses phenomenological reduction to reveal the underlying lived experiences and adaptive strategies of patients with lower limb lymphedema after gynecological cancer surgery. It not only enriches qualitative research in lymphedema care but also provides actionable theoretical and practical guidance for clinical nursing. The findings have important implications for optimizing postoperative rehabilitation pathways and improving patients’ quality of life—particularly in the context of increasingly prioritized patient-centered and holistic care models. This study establishes a solid theoretical foundation for developing precise and individualized nursing interventions. Future research should validate the proposed conceptual framework in larger and more diverse populations, and explore the integration of emerging technologies—such as artificial intelligence and telehealth into lymphedema management. Such efforts may facilitate a shift from reactive treatment to proactive prevention, ultimately enhancing both care quality and patient well-being.

## Data Availability

The original contributions presented in the study are included in the article/supplementary material, further inquiries can be directed to the corresponding authors.
